# Antiviral innate immunity in myocarditis: mechanisms and emerging therapeutics

**DOI:** 10.1038/s12276-026-01822-z

**Published:** 2026-09-03

**Authors:** Caleb Hammons, Athul Mohanram, Preston Nguyen, Wenting Lu, Betty Jacobs, Xian Chang Li, Zhiqiang Zhang, Junji Xing

**Affiliations:** 1 Immunobiology and Transplant Science Center, Department of Surgery, Houston Methodist Academic Institute, Houston Methodist, Houston, TX USA; 2 Department of Surgery, Weill Cornell Medicine, Cornell University, New York, NY USA; 3 Department of Cardiovascular Sciences, Houston Methodist Academic Institute, Houston Methodist, Houston, TX USA

**Keywords:** Infection, Innate immunity, Signal transduction

## Abstract

Antiviral innate immunity is a decisive determinant of whether cardiotropic viral infection resolves or progresses to myocarditis and deadly heart failure. Viral myocarditis (VM) remains a leading cause of sudden death in children and young adults, yet its human pathophysiological mechanisms remain incompletely understood and effective therapies are limited. In this Review, we map the cellular and molecular logic of cardiac antiviral innate immunity from first sensing to therapeutic opportunity and synthesize how these responses set the myocardial ‘inflammatory set point’ across cardiac-resident cells and recruited innate immune cells. We highlight RNA- and DNA-sensing immune signaling pathways that detect cardiotropic RNA and DNA viruses to collectively shape viral control and heart injury during VM. Finally, we connect mechanisms to translation by summarizing emerging biomarkers and clinical trials and by proposing a phase- and etiology-guided therapeutic framework that pairs antiviral immunomodulation with timely restraint of innate proinflammatory amplifiers to limit heart damage and prevent progression from VM to heart failure. This review will provide a roadmap of antiviral innate immunity to accelerate mechanistic discovery and targeted therapy for VM and virus-associated cardiovascular diseases.

## Mapping antiviral immunity in viral myocarditis progression

Myocarditis, an inflammatory heart disease, often progresses silently, making its true incidence hard to gauge. This Review consolidates recent insights into viral myocarditis, focusing on the role of innate immunity in its pathogenesis and potential therapeutic avenues. The authors explore how cardiotropic viruses trigger innate immune responses, involving both classical immune cells and cardiac-resident responders such as cardiomyocytes and fibroblasts. These cells detect viral nucleic acids, initiating inflammatory pathways that can lead to myocardial damage. Key findings highlight the dual role of innate immunity: while essential for antiviral defense, dysregulated responses can exacerbate tissue injury. The Review underscores the importance of understanding these mechanisms to develop targeted therapies, suggesting future directions such as pathway-specific immunomodulation and biomarker-driven diagnosis. This approach aims to refine treatment strategies, potentially improving outcomes for patients with viral myocarditis by preventing progression to chronic heart diseases.

This summary was initially drafted using artificial intelligence, then revised and fact-checked by the author.

## Introduction

Myocarditis is an inflammatory heart disease characterized by inflammatory immune cell infiltration of the myocardium and cardiomyocyte necrosis, which can progress to chronic dilated cardiomyopathy (DCM), heart failure and sudden death^1–5^. Because myocarditis is often asymptomatic and only approximately 10% of patients with clinical evidence are estimated to develop symptoms^5–7^, the true incidence of myocarditis is difficult to determine and often underestimated. Estimated incidence rates of myocarditis range from 0.12% to 12% according to various autopsy reports worldwide, or 4 to 14 per 100,000 people^5,8,9^. Global burden estimates from the Global Burden of Disease study suggest that myocarditis accounts for about 1.27 million incident cases worldwide in 2019, with the absolute number of cases increasing over recent decades^10^. Viral infections remain a leading cause of myocarditis worldwide, commonly referred to as viral myocarditis (VM).

VM is often conceptualized as a triphasic disease. The initial phase is driven by viral entry and replication in the heart, such as cardiomyocytes. This is followed by an immune activation phase in which innate and adaptive immune responses cooperate to limit viral spread but can also promote myocardial damage through excessive inflammation and cytotoxicity, including autoimmune-like mechanisms^11–13^. In the third phase, persistent injury and maladaptive repair contribute to fibrosis and ventricular remodeling, culminating in DCM^14–18^. Despite substantial progress from experimental models, the mechanisms that determine susceptibility, severity, and recovery in human VM remain incompletely understood, and effective and targeted therapies are still limited^19,20^. Although the acute phase of the coronavirus disease 2019 (COVID-19) pandemic has largely subsided, post-COVID syndromes (Long COVID) and related cardiovascular complications remain an ongoing clinical and public-health concern^21,22^. This continued burden of Long COVID underscores the need to deepen our understanding of the pathogenic mechanisms driving VM and to accelerate development of effective, mechanism-guided therapies for its prevention and treatment.

Innate immunity is the first line of host defense against virus infection^23,24^ and is increasingly recognized as a key determinant of outcome in VM^25–27^. Pattern-recognition receptors (PRRs) initiate type I interferon (IFN)-mediated antiviral responses while also activating proinflammatory pathways that, when dysregulated, can exacerbate tissue injury. Importantly, innate responses arise not only from classical immune cells but also from cardiac-resident populations such as cardiomyocytes and fibroblasts that can sense viral nucleic acids, propagate inflammatory signals and engage cell-death and stress pathways. These responses may also differ by sex, reflecting differences in innate signaling and immunoregulation that can influence clinical susceptibility and outcomes^3,12,28,29^.

In this Review, we consolidate recent advances linking antiviral innate immunity to VM pathogenesis and therapeutic opportunities. We first summarize the cardiotropic viruses implicated in VM and then discuss how classical innate immune cells and nonclassical cardiac-resident responders coordinate antiviral defense and inflammatory injury. Next, we focus on RNA- and DNA-sensing pathways and their downstream signaling networks, highlighting points of convergence that drive cytokine production, inflammasome activation and cytotoxic effector functions. Finally, we outline emerging translational directions, such as pathway- and cell-specific immunomodulation, and emphasize how integrating mechanistic insights with clinical correlates, such as candidate biomarkers of innate activation, may enable improved diagnosis, risk stratification and therapy.

## Etiology of VM

VM results from infection with a broad spectrum of cardiotropic viruses, and the dominant etiologic agents vary across age, geographic region, season and diagnostic modality^9,30^. Historically, enteroviruses, particularly coxsackievirus B3 (CVB3), have served as prototypical causes in experimental and clinical studies. However, contemporary series increasingly emphasize that the ‘leading’ viruses reported in VM may shift over time, in part because viral detection depends on sampling (for example, endomyocardial biopsy (EMB) versus blood), assay sensitivity and the timing of specimen collection relative to infection and immune clearance.

**Fig. 1:**
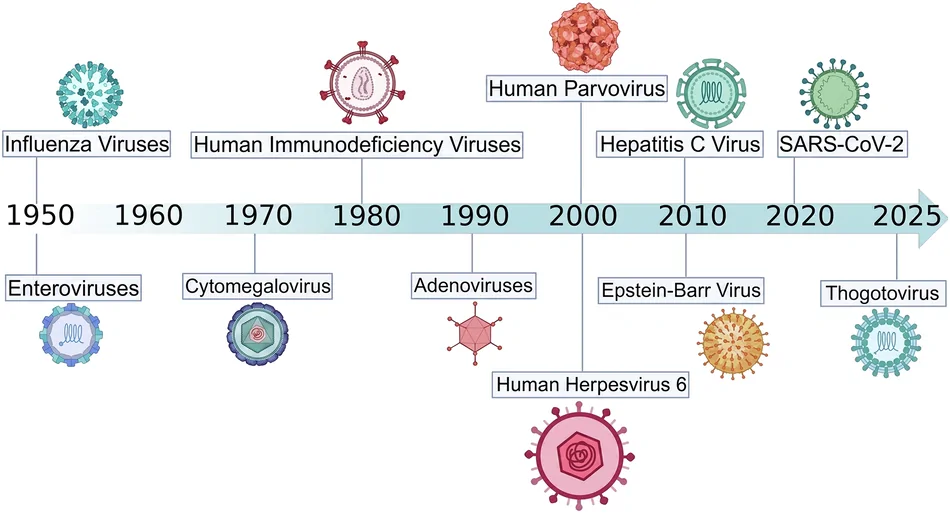
Timeline of discovery of major cardiotropic viruses causing VM. Schematic timeline illustrating the approximate decades in which key viruses relevant to VM were identified. Influenza viruses and enteroviruses were recognized in the 1950s; cytomegalovirus in the 1970s; human immunodeficiency viruses in the 1980s; adenoviruses in the 1990s; human parvovirus and human herpesvirus 6 in the 2000s; hepatitis C virus and Epstein–Barr virus in the 2010s; SARS-CoV-2 in 2020; and thogotovirus most recently in 2025.

Based on viral genome type, cardiotropic viruses can be broadly classified as RNA viruses and DNA viruses (Table 1). Typical cardiotropic RNA viruses (Fig. 1) include enterovirus CVB3^26,31–33^, influenza viruses^30,34–38^, human immunodeficiency virus (HIV)^30,39,40^, hepatitis C virus^30,41,42^ and other RNA viruses (Table 1), including enterovirus encephalomyocarditis virus (EMCV)^26,43–46^, reovirus^27,47–50^, ECHO viruses^51–53^ and respiratory syncytial virus (RSV)^54–56^. Typical cardiotropic DNA viruses (Fig. 1) include cytomegalovirus^30,33,57–59^, adenovirus^30,33,60,61^, parvovirus B19^30,62–64^, human herpesvirus 6^30,33,65,66^ and Epstein–Barr virus^30,33,66,67^ (Table 1). Importantly, VM can arise through multiple, nonmutually exclusive mechanisms: (1) direct viral infection of cardiomyocytes and/or cardiac-resident stromal cells; (2) innate immune activation driven by viral nucleic acids and damage-associated molecular patterns released from injured myocardium; and (3) downstream immune-mediated injury, including cytotoxic effector responses and autoimmune-like inflammation that may persist after the peak of viral replication.

**Table 1:** Cardiotropic viruses reported to cause VM.

Virus division	Virus names	Virus classification	Viral genome	References
RNA viruses	CVB3	Genus: *Enterovirus* Family: Picornaviridae	(+)ssRNA	^26,31–33^
	Influenza viruses	Genus: *Influenzavirus* Family: Orthomyxoviridae	(−)ssRNA	^30,34–38^
	HIV	Genus: *Lentivirus* Family: Retroviridae	(+)ssRNA	^30,39,40^
	Hepatitis C virus	Genus: *Hepacivirus* Family: Flaviviridae	(+)ssRNA	^30,41,42^
	EMCV	Genus: *Cardiovirus* Family: Picornaviridae	(+)ssRNA	^26,43–46^
	Reovirus	Genus: *Orthoreovirus* Family: Reoviridae	dsRNA	^27,47–50^
	ECHO viruses	Genus: *Enterovirus* Family: Picornaviridae	(+)ssRNA	^51–53^
	RSV	Genus: *Orthopneumovirus* Family: Pneumoviridae	(−)ssRNA	^54–56^
	SARS-CoV-2	Genus: *Betacoronavirus* Family: Coronaviridae	(+)ssRNA	^68–71^
	OZV	Genus: *Thogotovirus* Family: Orthomyxoviridae	(−)ssRNA	^76^
DNA viruses	Cytomegalovirus	Genus: *Cytomegalovirus* Family: Herpesviridae	dsDNA	^30,33,57–59^
	Adenovirus	Genus: *Mastadenovirus* Family: Adenoviridae	dsDNA	^30,33,60,61^
	Parvovirus B19	Genus: *Erythroparvovirus* Family: Parvoviridae	ssDNA	^30,62–64^
	Human herpesvirus 6	Genus: *Roseolovirus* Family: Herpesviridae	dsDNA	^30,33,65,66^
	Epstein–Barr virus	Genus: *Lymphocryptovirus* Family: Herpesviridae	dsDNA	^30,33,66,67^

(+)ssRNA, positive-sense single-stranded RNA; (−)ssRNA, negative-sense single-stranded RNA; ssDNA, single-stranded DNA.

Recent evidence also supports severe acute respiratory syndrome coronavirus 2 (SARS-CoV-2) as an infectious trigger for VM (Fig. 1 and Table 1) in some patients during the COVID-19 pandemic^68–75^. Although the relative contribution of direct myocardial infection versus systemic inflammation probably varies by clinical context, the COVID-19 era has reinforced the need to define how antiviral innate immune sensing and inflammatory circuits shape myocardial injury and outcomes. Moreover, continued viral emergence underscores etiologic breadth. For example, VM has recently been reported in association with Oz virus (OZV), a thogotovirus within the Orthomyxoviridae family^76^ (Fig. 1 and Table 1).

## Innate cells and innate immune responses in VM

Following infection with cardiotropic viruses, the earliest determinants of disease severity are often set by the magnitude, timing and cellular composition of the innate immune response. Innate immunity provides rapid antiviral defense, orchestrates leukocyte recruitment and shapes downstream adaptive responses; however, excessive or prolonged activation can drive cardiomyocyte injury, microvascular dysfunction and fibrotic remodeling. In VM, classical innate immune cells—including dendritic cells (DCs), macrophages, neutrophils, natural killer (NK) cells, natural killer T (NKT) cells and mast cells (MCs)—interact with cardiac-resident ‘nonclassical’ responders such as cardiomyocytes and cardiac fibroblasts (CFs). Together, these populations integrate viral sensing, cytokine production, cytotoxic effector functions and tissue repair programs, thereby influencing progression from acute myocarditis to chronic DCM and heart failure.

### Cardiac-resident innate responders

Cardiac-resident cells are not passive targets of infection. Cardiomyocytes and fibroblasts can detect viral nucleic acids, produce chemokines and cytokines and activate stress and cell-death programs that both limit viral spread and amplify inflammation. These responses can be beneficial early (antiviral restriction) but harmful when sustained (apoptosis, inflammatory amplification and fibrosis).

#### Cardiomyocytes

Cardiomyocytes are the contractile muscle cells of the heart and constitute the major cellular mass of the myocardium^77^. Beyond generating force through excitation–contraction coupling, cardiomyocytes are also active participants in cardiac innate immunity^78^. They express multiple PRRs and stress-response pathways, can produce cytokines and chemokines that recruit leukocytes, and can undergo regulated cell-death programs that shape both viral clearance and tissue injury^7^. Because many cardiotropic viruses directly infect cardiomyocytes, these cells sit at the intersection of direct viral cytotoxicity and host-driven inflammatory damage, making cardiomyocyte-intrinsic responses key determinants of disease severity and progression in VM.

During VM, cardiomyocytes can contribute to pathology through several interconnected mechanisms: (1) serving as targets for viral entry/replication; (2) initiating inflammatory signaling and chemokine production that amplifies immune-cell recruitment; and (3) activating cellular stress pathways that culminate in apoptosis or other forms of cell death, thereby increasing myocardial damage and promoting adverse remodeling. One cardiomyocyte-intrinsic pathway linked to VM involves the tripartite motif (TRIM) family protein TRIM29, which has been shown to engage the endoplasmic reticulum (ER) stress sensor protein kinase RNA-like ER kinase (PERK)^26^. PERK is activated by ER stress caused by accumulation of misfolded or unfolded proteins. Upon activation, PERK autophosphorylates and phosphorylates eukaryotic translation initiation factor 2α (eIF2α)^79^, attenuating global translation while selectively promoting stress-responsive transcriptional programs. Sustained PERK–eIF2α signaling induces C/EBP homologous protein (CHOP), a key mediator of pro-apoptotic signaling during prolonged ER stress. In CVB3-induced VM mouse model, TRIM29 has been reported to promote PERK recruitment and enhance PERK activation—at least in part by facilitating PERK SUMOylation—thereby amplifying PERK–eIF2α–CHOP signaling, increasing cardiomyocyte apoptosis and worsening myocardial injury and disease progression^26^.

These observations support a broader concept: cardiomyocyte stress pathways are not merely downstream consequences of inflammation but can actively shape VM outcomes. Therapeutically, cardiomyocyte-intrinsic regulators such as TRIM29 (pro-apoptotic/ER-stress-amplifying) represent potential targets to limit cardiomyocyte loss and adverse remodeling. Importantly, any intervention aimed at dampening cardiomyocyte death pathways should also take into account the preservation of antiviral defenses, as PRRs and stress responses in cardiomyocytes can also contribute to restricting viral replication and shaping protective immunity.

#### CFs

CFs are the principal stromal cells of the myocardium and the major source of extracellular matrix (ECM) proteins that provide the structural scaffold of the heart^80^. Under homeostatic conditions, CFs maintain a collagen-rich network that preserves tissue architecture, supports force transmission between cardiomyocytes and contributes to myocardial mechanical properties and electrical stability through cell–cell and cell–matrix interactions. CFs also serve as ‘sentinel’ cells that rapidly respond to tissue stress or injury by sensing pathogen-associated and damage-associated molecular patterns, thereby linking myocardial injury to inflammatory and reparative programs^81^.

In VM, CFs promote disease through three linked programs. First, they act as local inflammatory amplifiers: viral infection and cardiomyocyte injury activate CFs to secrete cytokines and chemokines (for example, TNF-α, IL-6 and MCP-1/CCL2) that recruit and instruct monocytes/macrophages and neutrophils, sustaining leukocyte infiltration. Second, CFs shape the myocardial microenvironment, modulating endothelial activation, leukocyte retention and local cytokine gradients to create feed-forward stromal–immune loops that may aid early antiviral coordination but become maladaptive when prolonged. Third, CFs drive remodeling and fibrosis by transitioning to myofibroblasts that deposit ECM, alter matrix composition and stiffness, and promote scar formation. Excessive or persistent activation leads to interstitial fibrosis, impaired contractility, arrhythmogenic substrate and adverse ventricular remodeling, facilitating progression toward DCM.

Notably, experimental work comparing CVB3 infection across murine cardiac cell types reported that CFs not only mount robust inflammatory responses (including increased TNF-α, IL-6 and chemokine production) but may also show susceptibility to viral infection and replication^82^. These findings raise the possibility that infected or activated CFs can serve as local sources of inflammatory mediators that amplify leukocyte recruitment and exacerbate myocardial injury. Together, the available evidence supports CFs as both inflammatory orchestrators and remodeling effectors in VM, suggesting that strategies aimed at tempering maladaptive CFs activation—while preserving necessary repair—may help to reduce chronic fibrosis and improve long-term outcomes.

### Classical innate immune cells

Classical innate immune cells are rapidly mobilized after cardiac infection. Their functions are phase dependent: early infiltration can restrict viral spread and prime adaptive immunity, whereas sustained activation can drive collateral heart damage through cytokines, reactive oxygen species, proteases and cytotoxic programs. The balance between protective antiviral activity and immunopathology is therefore a central theme in VM.

#### NK cells

NK cells are innate lymphocytes that provide rapid, antigen-independent cytotoxicity and cytokine production during the earliest phase of viral infection^83^. Unlike T cells, NK cells do not require prior sensitization to a specific viral peptide–MHC complex. Instead, they integrate signals from activating and inhibitory receptors to detect ‘altered-self’ states in infected or stressed cells, such as reduced MHC class I expression (‘missing-self’) and increased expression of stress-induced ligands (‘induced-self’). Because these host-cell changes occur after infection by many unrelated pathogens, NK cells can respond broadly to both RNA and DNA viruses, making them a front-line effector population in VM across diverse etiologies^84^.

In VM, NK cells kill virus-infected cardiomyocytes to limit virus dissemination^85^. We previously demonstrated that TRIM29 suppresses IFN-γ production in NK cells, thereby facilitating murine cytomegalovirus infection^86^. Consistent with a protective role, deficiency or depletion of NK cells in mouse models is associated with higher cardiac viral titers and increased myocardial injury^87^, supporting the idea that NK cells contribute to early containment of cardiotropic viral infection. However, NK cells can also promote immunopathology when activation is excessive or prolonged. Overactivation and/or sustained accumulation of NK cells in the myocardium may increase cardiomyocyte loss through release of cytotoxic granules and inflammatory mediators. In line with this, reduced perforin activity can attenuate tissue injury in EMCV-induced mouse model^88^, underscoring that NK-mediated cytotoxicity—while antiviral—can be a major driver of myocardial necrosis and subsequent remodeling if not properly regulated.

**Fig. 2:**
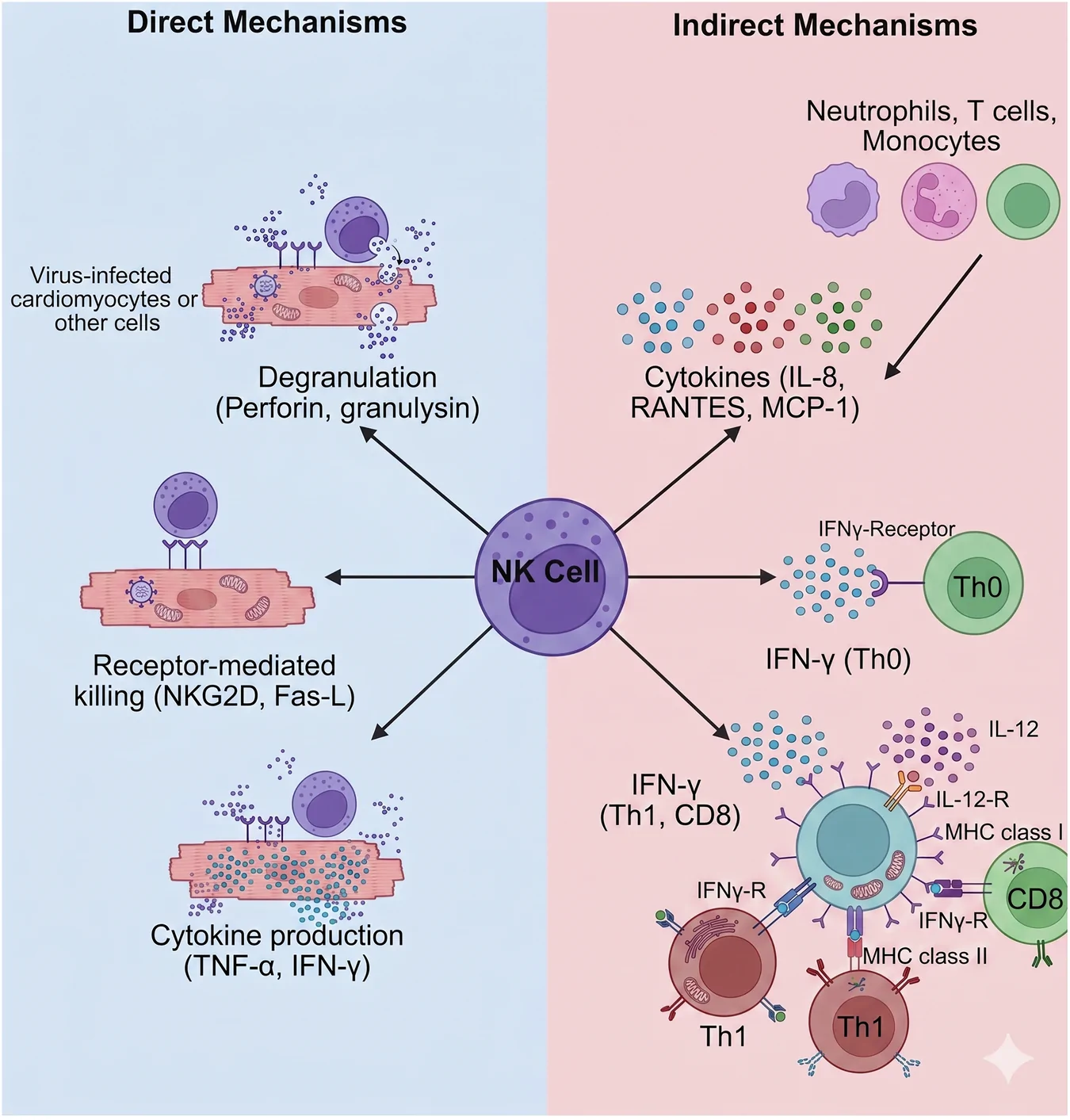
Direct and indirect antiviral effector mechanisms of NK cells. Schematic illustration of the direct and indirect pathways by which NK cells contribute to antiviral immunity. Direct mechanisms include cytotoxic degranulation (perforin and granulysin), receptor-mediated killing of virus-infected cardiomyocytes or other target cells (for example, via NKG2D and Fas ligand) and production of antiviral cytokines such as IFN-γ and TNF. Indirect mechanisms involve NK cell-derived cytokines and chemokines that recruit and activate other immune cells, including neutrophils, monocytes and T cells, and promote adaptive immune responses through polarization of Th1 cells and activation of CD8^+^ T cells.

Mechanistically, NK cells exert antiviral activity in VM through several complementary pathways. First, direct cytotoxic killing occurs through granule exocytosis of perforin and granzymes, inducing apoptosis in infected cardiomyocytes. NK cells also use death receptor pathways (for example, FasL/Fas and TRAIL/TRAIL receptors) to trigger apoptosis in target cells. Second, receptor-mediated recognition amplifies killing: viral infection and cellular stress increase expression of ligands for activating receptors such as NKG2D, promoting activation of NK cells and target-cell lysis. Third, cytokine-mediated antiviral effects are critical. NK cells produce IFN-γ and TNF-α, which suppress viral replication, enhance antigen presentation and shape downstream immune responses; they can also produce immunoregulatory cytokines such as IL-10, which may limit collateral tissue damage in some settings. Activation of NK cells is strongly driven by innate cytokines (for example, IL-12, IL-15 and IL-18) released from DCs, macrophages and other tissue-resident cells after viral sensing, positioning NK cells as a key bridge between early PRRs signaling and broader immune orchestration. Beyond direct antiviral control, NK cells influence the transition to adaptive immunity. IFN-γ promotes effector maturation of NK cells and can enhance both direct effector functions (degranulation and cytokine production) and indirect functions, including promoting DC maturation and facilitating Th1-polarized adaptive responses (Fig. 2).

Conversely, NK cells also shape inflammatory tone and tissue injury. For example, depletion of NK cells has been linked with more severe myocarditis and altered regulation of eosinophilic responses^89^, suggesting context-dependent roles in controlling immunopathology. Host factors further modulate impact of NK cells during VM. FOXO3 has been identified as a negative regulator of NK cells antiviral activity; mice lacking FOXO3 exhibited lower cardiac viral loads^90^, consistent with enhanced NK-mediated viral control. Sex hormones also influence NK cells responses: estrogen can dampen IFN-γ production, and reduced estrogen has been associated with worsened myocarditis in females^91^, providing a plausible mechanistic link between sex differences and NK-driven inflammatory balance.

Overall, NK cells are double-edged effectors in VM: early on, well-regulated NK responses can restrict viral replication and shape protective immunity, whereas excessive cytotoxicity or prolonged activation can amplify cardiomyocyte loss and exacerbate myocardial injury. Therapeutic strategies that preserve early antiviral NK functions while limiting sustained cytotoxic damage—potentially by timing interventions or targeting specific activation axes (for example, NKG2D signaling)—may be particularly relevant for preventing progression from acute VM to chronic DCM and heart failure.

#### DCs

DCs are professional antigen-presenting cells that bridge innate sensing, tissue inflammation and adaptive immunity^27,92–95^. Rather than functioning as a single uniform population, DCs comprise specialized subsets with distinct sensing and instructional roles^96^. The cDC1 cells are specialized for cross-presentation of exogenous antigens on MHC class I and efficient priming of cytotoxic CD8^+^ T cells^97^, supporting antiviral immunity and elimination of virus-infected cells. In the heart, CD103^+^ cDC1-mediated surveillance protects against progression of subclinical VM^98^. The cDC2 cells preferentially present antigen through MHC class II to CD4^+^ T cells and shape their polarization toward inflammatory Th1 or Th17 effector programs or, in regulatory contexts, toward regulatory T (T_reg_) cells that restrain inflammation and promote resolution^97^. In myocarditis, cDC2 cells can also present cardiac self-antigens released during tissue injury, potentially promoting pathogenic autoreactive CD4^+^ T cell responses and autoimmune-like myocardial inflammation when tolerance mechanisms fail^99^. Plasmacytoid DCs serve as rapid and robust producers of type I IFN following endosomal sensing of viral nucleic acids, providing an early antiviral shield that limits initial viral replication in the heart but, if dysregulated or prolonged, may drive excessive inflammation or break self-tolerance^100,101^.

These subset-specific functions are particularly relevant in VM because disease outcome depends on whether DCs promote protective antiviral priming or excessive autoreactive and inflammatory responses. Recent studies in human myocarditis and experimental myocarditis models demonstrate prominent monocyte and DCs infiltration into the inflamed heart and underscore the need to define DCs subset composition, tissue localization and functional specialization during early myocarditis^99,102,103^. In VM, DCs can amplify disease by presenting viral and cardiac antigens, providing costimulatory signals (for example, CD80, CD86 and CD40) and producing cytokines (for example, IL-12, IL-23 and TNF-α) that promote inflammatory T cell differentiation and effector functions. At the same time, DCs can limit disease by inducing robust antiviral type I IFN responses, supporting controlled viral clearance and maintaining peripheral and central tolerance to self-antigens through mechanisms such as PD-L1 expression or T_reg_ cell induction. Cardiac DCs also engage in extensive crosstalk with the local tissue microenvironment. Interactions with cardiomyocytes may involve direct antigen presentation or cytokine feedback loops that influence cardiomyocyte survival and stress responses^26^; communication with CFs can reinforce chemokine gradients (for example, CCL2 and CXCL10) and leukocyte recruitment while modulating CF activation and ECM production^104^; crosstalk with endothelial cells upregulates adhesion molecules (for example, ICAM-1 and VCAM-1) to facilitate leukocyte extravasation^104^; and coordination with macrophages shapes the balance between inflammatory and reparative states^96,105,106^. For example, a CD103^+^ cDC1 surveillance system has been reported to restrain overt heart failure during subclinical VM^98^, supporting a protective role for specific DCs subsets in maintaining cardiac immune balance.

Beyond canonical antigen presentation and subset functions, DCs contribute to VM through innate nucleic acid sensing and type I IFN production. As one illustrative example of emerging DC-intrinsic antiviral sensing within the broader framework of canonical DCs biology, we identified poly(ADP-ribose) polymerase 9 (PARP9) as a noncanonical, mitochondrial antiviral-signaling (MAVS)-independent RNA sensor in DCs that binds viral double-stranded RNA (dsRNA) and promotes type I IFN responses^27^. Mechanistically, PARP9–dsRNA engagement triggers recruitment of phosphoinositide 3-kinase (PI3K) and activation of the PI3K–AKT3 axis, leading to phosphorylation and activation of interferon regulatory factor 3 (IRF3)/IRF7 and subsequent induction of type I IFN independently of MAVS^107^. This pathway exemplifies how emerging DC-specific viral sensing mechanisms can complement canonical RNA-sensing pathways rather than sensing as the dominant focus. Future single-cell, spatial and lineage-tracing approaches will be needed to determine how cDC1, cDC2, plasmacytoid DCs and monocyte-derived DC states are distributed across VM stages and how they influence cardiomyocyte survival, instruct CF activation, modulate macrophage-driven inflammation and shape T cell pathogenicity within the evolving cardiac microenvironment.

#### Macrophages

Macrophages are among the dominant innate immune populations in inflamed myocardium and participate in viral sensing, phagocytosis, cytokine production, antigen presentation, efferocytosis and tissue repair^106,108^. In CVB3-induced VM, macrophages constitute a major fraction of infiltrating leukocytes and can either restrict viral replication or amplify myocardial injury. Earlier VM studies often classified macrophages into M1 and M2 states^109^, a useful but oversimplified framework. Susceptible male BALB/c mice show inflammatory macrophage programs enriched for Th1-associated cytokines and chemokines, whereas females more often show alternatively activated and reparative features; moreover, transfer of in vitro-polarized M2-like macrophages into male mice reduces myocarditis severity, supporting the concept that macrophage state strongly influences disease outcome^109^. Contemporary single-cell and spatial transcriptomic studies show that cardiac macrophages instead occupy a spectrum of states shaped by ontogeny, tissue niche, viral load, cytokines and time after injury. These include resident cardiac CCR2^−^ macrophages^110–112^, recruited CCR2^+^ monocyte-derived inflammatory macrophages^109,111,113^, IFN-stimulated macrophages^114–116^, antigen-presenting macrophages^117–120^ and reparative/profibrotic macrophages^121–125^ states. These distinctions are important for therapeutic translation because broad macrophage depletion may remove both pathogenic and protective subsets^106,126,127^. Thus, the key issue in VM is not simply whether macrophages are M1 or M2, but which macrophage states dominate at a given disease phase and where they are positioned within the injured myocardium. To improve clarity, we explicitly link these macrophages states below to disease phase, tissue niche, dominant functions and outcome in VM (Table 2).

**Table 2:** Phase- and niche-linked macrophage states in VM.

Macrophage state	Disease phase	Primary tissue niche	Dominant functions	Likely outcome in VM
Resident cardiac CCR2^−^ macrophages	Homeostasis and early acute phase	Myocardial interstitium, perivascular regions and epicardial niches	Support electrical conduction, clear apoptotic cells by efferocytosis, maintain tissue homeostasis and produce anti-inflammatory or tissue-supportive mediators	Generally protective: limits excessive inflammation, preserves myocardial function and supports electrical stability^110–112^
CCR2^+^ monocyte-derived inflammatory macrophages	Acute inflammatory peak	Areas of active viral replication, cardiomyocyte necrosis and inflammatory border zones	Produce proinflammatory cytokines, including TNF-α, IL-1β and IL-6; secrete chemokines such as CCL2 and CXCL10; generate reactive oxygen species; and phagocytose debris	Dual-edged: contributes to viral and debris clearance but can amplify edema, cardiomyocyte injury and inflammatory tissue damage if unresolved^109,111,113^
IFN-stimulated macrophages	Early antiviral phase	IFN-rich regions near infected cardiomyocytes and inflamed myocardium	Upregulate IFN-stimulated genes (ISGs), enhance antigen-presentation machinery and reinforce antiviral cytokine programs	Protective when timely and transient, but prolonged activation may sustain inflammatory signaling^114–116^
Antigen-presenting macrophages	Subacute immune-priming phase	Perivascular regions, T cell-rich inflammatory foci and sites near draining lymphatic routes	Process and present viral and cardiac antigens through MHC I/II, provide costimulatory signals, and shape Th1/Th17 versus regulatory T cell responses	Supports adaptive viral clearance but may contribute to pathogenic T cell activation and autoimmune-like cardiac injury if tolerance fails^117–120^
Reparative/profibrotic macrophages	Resolution, late acute phase and chronic remodeling	Fibrotic border zones adjacent to activated CFs	Promote efferocytosis through receptors such as MerTK, produce IL-10 and TGF-β, support angiogenesis, interact with CFs and regulate ECM remodeling	Facilitates inflammation resolution and wound repair, but excessive or persistent activation may promote fibrosis, ventricular stiffening and progression to DCM^121–125^

Macrophages also integrate antiviral nucleic acid sensing with downstream remodeling programs. For example, macrophage-intrinsic signaling through type I IFN production pathways, inflammasomes and cytokine receptors can tune the balance between type I IFN-mediated viral restriction and heart injury driven by proinflammatory cytokines IL-1β, TNF-α and IL-6. E3 ubiquitin ligase TRIM18 has been reported to recruit protein phosphatase 1A (PPM1A) to dephosphorylate TANK-binding kinase 1 (TBK1), thereby dampening RNA sensing and MAVS-dependent type I IFN signaling and increasing the severity of CVB3-induced cardiac inflammation^116^. Future work should move beyond bulk macrophage markers and use single-cell multi-omics, spatial transcriptomics and fate mapping to determine which macrophage states are protective, pathogenic or reparative in VM, and how these states communicate with cardiomyocytes, CFs, endothelial cells and adaptive immune cells during progression to DCM and heart failure.

#### Neutrophils

Neutrophils are short-lived, bone marrow–derived granulocytes and the most abundant circulating leukocytes in humans^128^. As rapid ‘first responders’ of innate immunity, they are recruited within minutes to hours to sites of infection or sterile tissue injury, where they eliminate viruses through phagocytosis, degranulation of antimicrobial proteins, generation of reactive oxygen species and formation of neutrophil extracellular traps (NETs)^129^. Through cytokine and damage-associated molecular pattern signaling by damaged cardiomyocytes, macrophages and endothelial cells, neutrophils are recruited and migrate to sites of damaged or inflamed cardiac cells^30,130,131^. Following activation, neutrophils release anti-microbial proteins such as defensins or proteases, and release proinflammatory cytokines that amplify the inflammatory response^132^. Artificially induced neutrophil depletion before the acute phase of VM reduces cardiac monocyte recruitment, resulting in decreased levels of proinflammatory cytokines and chemokines^133^. This finding indicates that the recruitment of proinflammatory leukocytes can induce a harmful elevated inflammatory response in cardiac tissue during VM. Neutrophils also produce NETs, which are composed of decondensed DNA complexed with antimicrobial proteins that immobilize and destroy pathogens^134^. DNA decondensation is mediated by peptidyl arginine deaminase 4 (PAD4), which converts histone arginine residues to citrulline, weakening histone–DNA binding. PAD4-deficient mice are unable to form NETs in response to pathogens, such as infectious bacteria^135,136^. In addition, PAD4-knockout mice showed lower monocyte presence, low proinflammatory signals and a reduction in cardiac necrosis in both early and late acute VM^133^, suggesting that the formation of NETs are detrimental to cardiac tissue during acute VM.

#### NKT cells

NKT cells are innate-like T lymphocytes that share features of both conventional T cells and NK cells^137^. They express a T cell receptor but, unlike conventional T cells, many NKT cells recognize lipid antigens presented by CD1d. NKT cells respond rapidly after activation, producing large amounts of cytokines and occasionally exerting cytotoxic activity^95^. Invariant NKT (iNKT) cells are the best-studied subset of NKT cells and can regulate early immune responses^138^.

In VM, iNKT cells can shape disease by rapidly tuning the cytokine milieu and downstream innate/adaptive immunity. Several studies suggest that iNKT cells may be protective, limiting excessive inflammation in a T_reg_-like manner while supporting antiviral defense^139–141^. However, findings in CVB3-induced VM are mixed: some reports support a beneficial role, whereas others observe increased iNKT cells during acute VM and an association with inflammatory severity^142^. These differences probably reflect variation in timing, viral strain/dose and host factors.

#### MCs

MCs are long-lived, tissue-resident innate immune cells best known for their roles in allergic inflammation, but they also participate broadly in host defense and sterile tissue injury^95,143^. In VM, experimental evidence indicates that MCs activation occurs early after cardiotropic viral infection and can influence both acute inflammatory injury and later ventricular remodeling. In a CVB3-induced VM model comparing susceptible and resistant mouse strains, Fairweather et al. showed that susceptibility to autoimmune-like heart disease is established very early after infection. Susceptible BALB/c mice exhibited prompt increases in TNF-α, IL-1β and IL-4, and degranulation of MCs was observed in the heart as early as about 6 h after infection, accompanied by higher splenic MC numbers, suggesting that early MC-driven cytokine programs may bias subsequent immunopathology^144^. Two complementary studies in an EMCV-induced VM mouse model further link MCs to myocardial remodeling and fibrosis. Kitaura-Inenaga et al. reported upregulation of MCs proteases—chymase (mMCP-4/5) and tryptase (mMCP-6)—from day 5 through day 14, coinciding with inflammation, necrosis and fibrosis, and correlating with increased MMP-9 and type I procollagen, indicating that fibrotic programs begin during the acute stage^145^. Providing causal evidence, Higuchi et al. demonstrated that two distinct MC-deficient strains had improved survival and markedly reduced necrosis and inflammatory infiltration, whereas MC reconstitution worsened pathology. Pharmacologic attenuation with an H1 receptor antagonist (bepotastine) improved disease, supporting MC effector pathways as therapeutic targets^45^. Together, these studies suggest that MCs contribute to VM by (1) initiating early cytokine signals that shape susceptibility and (2) promoting remodeling through protease- and MMP-linked matrix turnover. Future work should define virus- and context-specific mechanisms and identify intervention windows that limit MC-mediated injury while preserving antiviral protection.

### Crosstalk between innate and adaptive immunity

Innate and adaptive immunity are tightly intertwined during VM, and their bidirectional crosstalk largely determines whether the host achieves viral clearance with tissue recovery or progresses to persistent inflammation and adverse remodeling. Early after infection, cardiac-resident cells and infiltrating innate leukocytes sense viral nucleic acids and tissue damage and generate a local ‘instructional’ milieu composed of type I IFN, inflammatory cytokines and chemokines. These signals shape antigen handling, antigen presentation and T cell priming, thereby linking early innate recognition to downstream adaptive effector programs.

Professional antigen-presenting cells—especially DCs and macrophages—capture viral antigens (and, in some contexts, cardiac self-antigens released from injured cardiomyocytes), process them and present peptides on MHC molecules to T cell receptors. Productive priming requires both antigen presentation and costimulatory signals, leading to activation and expansion of antigen-specific T lymphocytes and, secondarily, B cell responses^146^. The resulting adaptive immunity is essential for pathogen elimination: cytotoxic CD8^+^ T cells can kill infected cardiomyocytes, CD4^+^ T cells provide help for antibody production and orchestrate inflammation, and B cells generate neutralizing antibodies that limit viral spread. Recently, we showed that TRIM29 deficiency reduces PERK-mediated immunosuppressive monocytic myeloid-derived suppressor cells to enhance functions of antiviral CD8^+^ T cells for killing CVB3 during VM in vivo^26^. However, these same adaptive programs can also become pathogenic. Excessive or prolonged T cell activation amplifies inflammatory cytokine production and cytotoxic injury, and may promote epitope spreading or autoimmune-like responses to cardiac antigens, thereby sustaining myocardial inflammation even after viral titers decline^147,148^.

Crosstalk is also bidirectional: adaptive immune cells feedback to reshape innate responses within the myocardium. Activated T cells can promote recruitment and polarization of monocytes/macrophages and enhance inflammatory activation of endothelial cells and CFs, increasing leukocyte retention and local cytokine gradients. Conversely, innate-cell-derived cytokines critically tune adaptive polarization. Thus, VM can be viewed as a dynamic immune circuit in which innate signals set the initial trajectory of adaptive immunity, and adaptive responses subsequently reinforce—or restrain—local innate inflammation and remodeling. Importantly, neuroimmune regulatory pathways can modulate this innate–adaptive axis. The α7 nicotinic acetylcholine receptor (α7nAChR), a key component of the cholinergic anti-inflammatory pathway, has been reported to reduce proinflammatory cytokine production and suppress inflammatory signaling in VM, highlighting a mechanism by which immune activation may be dampened without completely abrogating antiviral defense^149^. Together, these findings underscore that effective therapies need to preserve early protective antiviral responses while selectively limiting maladaptive innate–adaptive amplification that drives chronic heart inflammation.

Taken together, this framework highlights two therapeutic inflection points: (1) early antiviral defense, where strengthening protective IFN programs and coordinated innate responses may limit viral expansion, and (2) immune-mediated injury/remodeling, where selectively dampening maladaptive inflammation may prevent progression to chronic cardiomyopathy without compromising viral control. The following sections focus on the upstream molecular ‘wiring’ that links viral nucleic acids to these cellular outcomes—specifically RNA- and DNA-sensing pathways. We emphasize how these pathways regulate IFN induction, inflammatory cytokine programs and inflammasome activation, and how their timing and cell-type specificity ultimately determine myocardial injury versus repair.

### Gut–heart immune axis in VM

The gut–heart immune axis is an emerging framework for understanding VM, especially for enteric cardiotropic viruses such as CVB3. Because CVB3 initially replicates in the gastrointestinal tract before systemic dissemination, mucosal barrier integrity, intestinal innate immune tone and commensal microbiota can influence viral burden, systemic cytokine responses and subsequent cardiac inflammation. The gut microbiome educates innate and adaptive immunity through microbial-associated molecular patterns, metabolites such as short-chain fatty acids, bile-acid derivatives and tryptophan metabolites, and tonic regulation of epithelial and myeloid-cell IFN responses. These signals can prime the systemic inflammatory ‘set point’, altering monocyte/macrophage activation, neutrophil recruitment, NK cell function and T cell polarization before or during cardiac infection^150,151^.

Experimental and clinical observations suggest that intestinal factors play a role in VM susceptibility. CVB3 infection can alter gut microbial composition and metabolic profiles, and intestinal immune sites may influence systemic viral dissemination and cardiac inflammation. For example, appendectomy was reported to reduce intestinal and systemic viral burden and alleviate CVB3-induced myocarditis in mice, suggesting that intestinal immune niches may contribute to the magnitude of downstream cardiac inflammation^152^. However, the gut–heart axis remains mechanistically underdeveloped in VM. Key future questions include whether specific microbial communities or metabolites enhance antiviral type I IFN without promoting immunopathology, whether dysbiosis increases epithelial permeability and systemic inflammatory priming, and whether microbiome-directed interventions can reduce progression from acute VM to chronic DCM. Integrating gut microbiome profiling with cardiac single-cell/spatial immune maps may therefore provide a more holistic view of VM pathogenesis and reveal modifiable upstream determinants of the cardiac inflammatory set point.

## RNA-sensing pathways in antiviral innate immunity during VM

RNA-sensing PRRs are central to the early host response against cardiotropic RNA viruses. These sensors detect viral RNA in distinct subcellular compartments—endosomes (for example, TLR3/7/8) and the cytosol (for example, RIG-I–like receptors)—and activate signaling cascades that converge on type I IFN and proinflammatory cytokines programs. In VM, these pathways play a dual role: they are essential for limiting viral replication and dissemination, yet excessive or prolonged activation can amplify immune-mediated myocardial injury. Below, we summarize major RNA-sensing pathways and highlight how compartmentalization, adaptor usage and host context (for example, sex) influence disease outcomes.

### Toll-like receptors (TLRs)

**Fig. 3:**
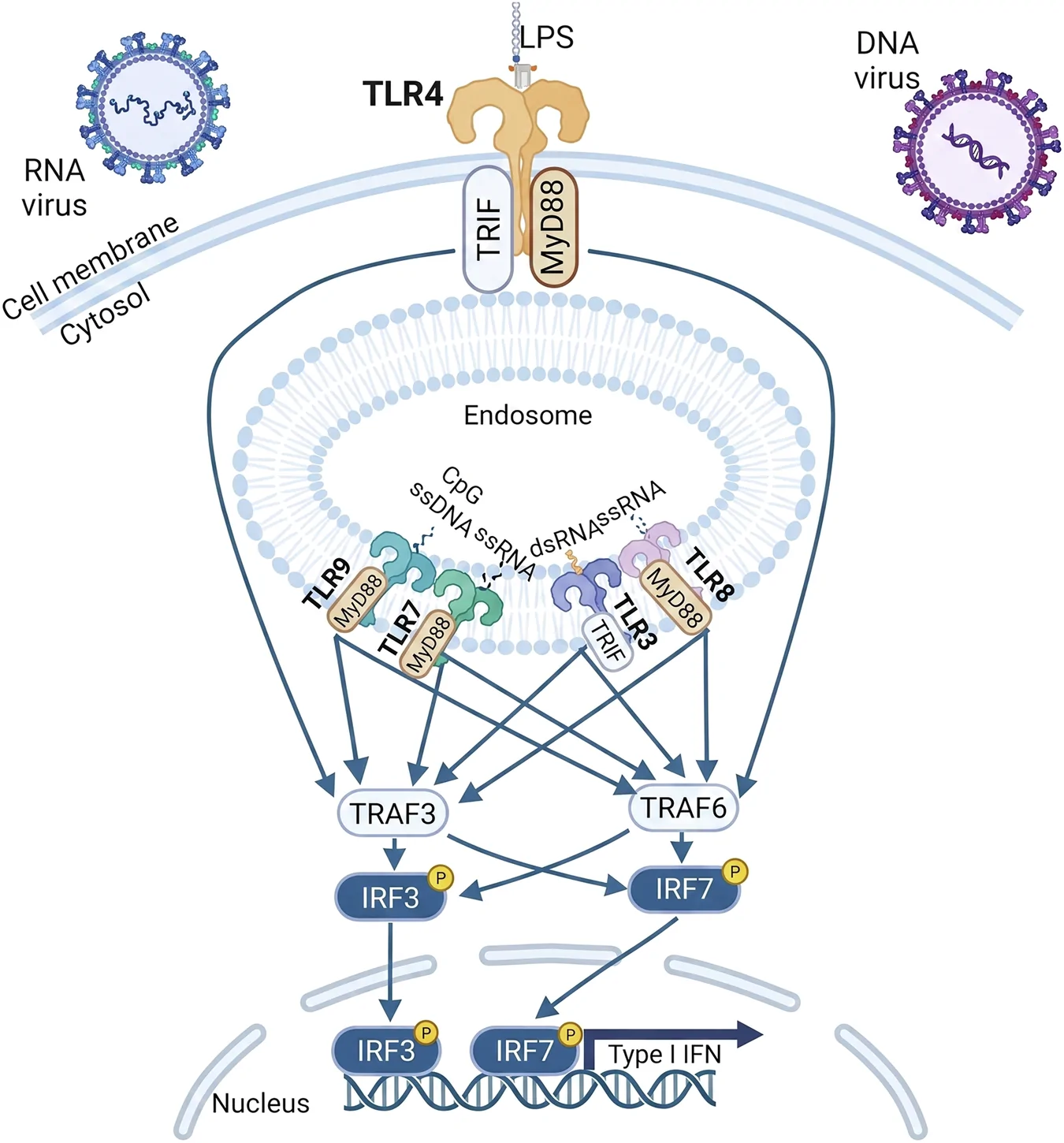
TLR signaling pathways in antiviral innate immunity during VM. Schematic representation of TLR-mediated innate immune signaling in response to viral and microbial PAMPs during VM. Endosomal TLR3 senses viral dsRNA and signals through the adaptor TRIF, whereas TLR7 and TLR8 detect single-stranded RNA (ssRNA) and TLR9 recognizes CpG-containing single-stranded DNA (ssDNA), all primarily via the adaptor MyD88. Cell-surface TLR4 responds to lipopolysaccharide (LPS) and signals through both TRIF- and MyD88-dependent pathways. Engagement of these TLRs converges on activation of the adaptor proteins TRAF3 and TRAF6, leading to phosphorylation and activation of the transcription factors IRF3 and IRF7. Activated IRF3 and IRF7 translocate to the nucleus to induce type I IFN gene expression, promoting antiviral immune responses in the heart.

TLRs are germline-encoded PRRs that detect pathogen-associated molecular patterns (PAMPs) and initiate innate immune responses. In mammals, 13 TLRs have been identified, including 10 in humans^153^. TLRs are highly expressed in professional innate immune cells (for example, DCs and macrophages) but are also present at lower levels in non-immune cardiac cells. Among them, TLR3 and TLR7/8 are key RNA sensors: TLR3 recognizes viral dsRNA, whereas TLR7/8 recognize ssRNA within endosomes^154,155^ (Fig. 3). Although TLR2, TLR4 and TLR9 are not canonical RNA sensors, they can be engaged during viral infection through viral proteins, host-derived damage signals or co-infections, therefore modulating inflammation in VM.

#### Adaptor usage: TRIF versus MyD88

Following ligand recognition, TLRs signal through adaptor molecules—primarily MyD88 or TRIF—to activate NF-κB and IFN-regulatory factors (IRFs), leading to inflammatory cytokine production and IFN-stimulated gene induction^156^. In CVB3 infection, genetic evidence indicates that TRIF-dependent signaling is particularly important for TLR3-driven protection: MyD88 appears less essential for the TLR3 arm than TRIF in this setting^157,158^. Consistent with this, TRIF-deficient mice infected with CVB3 exhibit higher viral titers, worse cardiac dysfunction and increased early mortality^159^. Mechanistically, TRIF promotes IRF activation (notably IRF7 in the TLR3/TLR4 axis), supporting antiviral IFN programs^160–162^ (Fig. 3). By contrast, MyD88 is the dominant adaptor for most other TLRs (for example, TLR7, TLR8 and TLR9), while TLR4 can signal through both MyD88 and TRIF (Fig. 3).

#### TLR3: protective signaling beyond classic type I IFN

Multiple studies support a protective role for TLR3 in enteroviral myocarditis. TLR3 deficiency increases susceptibility to CVB3/CVB4 infection, with higher mortality and more severe myocarditis^158,163^. Interestingly, some studies report minimal effects of TLR3 deficiency on type I IFN mRNA, suggesting that TLR3-mediated protection may not be explained solely by type I IFN induction^164^. Instead, TLR3 has been linked to broader antiviral programs, including pathways intersecting with type II IFN responses, which may help explain its protective effect even when type I IFN signatures are not strongly altered^165^.

#### TLR7/8: ssRNA sensing and cytokine amplification

TLR7/8 can be strongly induced after exposure to enteroviral ssRNA. In vitro, exposure to CVB3 ssRNA upregulates TLR7/8 and drives inflammatory cytokine production in a TLR7/8-dependent manner^155,166^. Functionally, TLR7 signaling promotes type I IFN and proinflammatory cytokines such as IL-12^167^. In transfected HEK cell systems, TLR8 has been reported to elicit particularly robust IL-6 responses to CVB3, and both TLR7 and TLR8 can promote production of IL-6, TNF-α and IFN-β after exposure to CVB3 virions^166^. These findings suggest that the TLR7/8 axis can amplify inflammatory cytokine programs—potentially beneficial for antiviral defense but capable of worsening immunopathology if excessive^166,168^.

#### TLR4 and sex-specific immunopathology

Although classically considered an LPS receptor, TLR4 is activated during CVB3 infection and can promote myocardial inflammation^169,170^. TLR4 deficiency is associated with reduced myocardial inflammation in CVB3-infected mice^171^. Notably, TLR4-associated pathogenicity can be sex-biased: male mice show higher TLR4 induction after CVB3 infection than females, whereas females may show higher TLR2 responses^172^. This difference is relevant because TLR2 signaling can confer relative protection while TLR4 signaling tends to increase pathogenicity in this model^172^. Consistent with these observations, females often exhibit less severe acute inflammation in CVB3-induced VM^173^. Tim-3 has also been implicated in sex-biased regulation of inflammatory responses, with Tim-3 blockade in males increasing inflammation and TLR4^174^. Together, these data highlight that TLR-driven programs are context dependent and influenced by host factors such as sex.

While endosomal TLRs sense extracellular or endocytosed viral RNA, many cardiotropic RNA viruses replicate in the cytosol. Accordingly, cytosolic RNA helicases provide an additional layer of viral detection that is particularly relevant to infected cardiomyocytes.

### RIG-I-like receptors (RLRs)

**Fig. 4:**
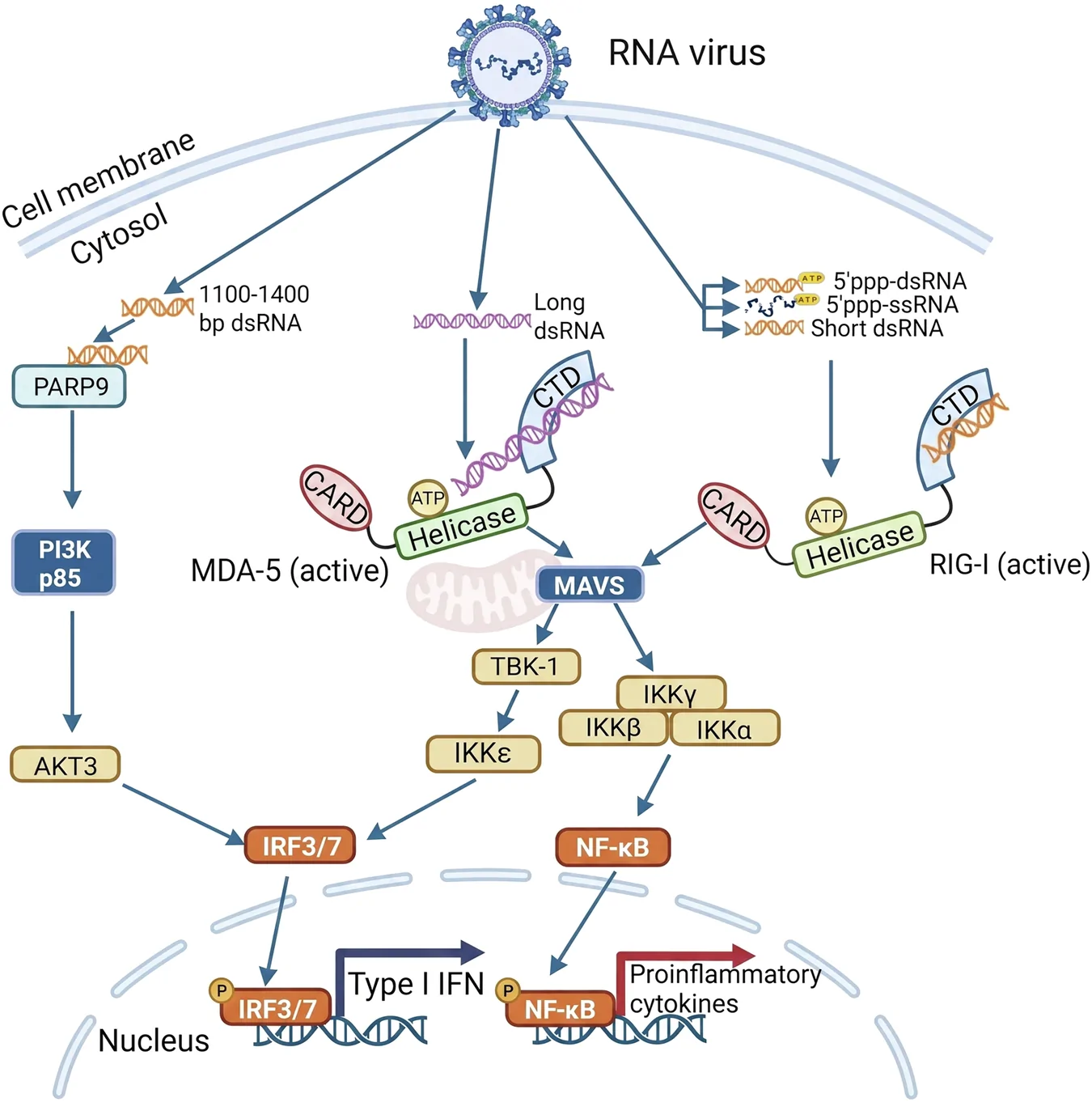
RNA-sensing pathways in antiviral innate immunity during VM. Schematic overview of RLR-mediated and noncanonical RNA-sensing pathways activated by cardiotropic RNA viruses during VM. Viral RNA is recognized by RIG-I or MDA5 through binding to their C-terminal domains (CTDs), inducing conformational changes that expose the caspase activation and recruitment domains (CARDs). CARD–CARD interactions promote activation of the mitochondrial adaptor MAVS, leading to downstream signaling through TBK1 and IKK complexes, activation of IRF3 and IRF7, and induction of type I IFN, as well as activation of NF-κB and production of proinflammatory cytokines. In parallel, viral dsRNA can be sensed by the noncanonical RNA sensor PARP9, which activates a PI3K–AKT3 signaling axis to induce IRF3/7 activation independently of MAVS, further contributing to antiviral immune responses in the heart. Activating signals are shown with blue arrows; type I IFN transcription, which restricts VM, is indicated by a bold blue arrow, whereas transcription of proinflammatory cytokines, which promotes VM, is indicated by a bold red arrow.

RLRs detect viral RNA in the cytosol, complementing endosomal TLR sensing. CVB3 RNA and replication intermediates are present in cytoplasmic compartments after infection, supporting a role for cytosolic sensors in cardiomyocytes^175^. The major RLRs are retinoic acid-induced protein-I (RIG-I) and melanoma differentiation-associated gene 5 (MDA5)^176,177^, which recognize distinct viral RNA features and signal through the adaptor MAVS (Fig. 4).

#### Ligand specificity and virus preference

RIG-I and MDA5 exhibit differences in ligand preference and viral selectivity. RIG-I responds strongly to shorter dsRNA and 5′-triphosphorylated ssRNA (5′ppp-ssRNA), whereas MDA5 is preferentially activated by longer dsRNA (for example, high-molecular-weight poly I:C)^178^. In terms of viral selectivity, RIG-I recognizes many RNA viruses (for example, rhabdoviruses, orthomyxoviruses and paramyxoviruses)^179,180^, whereas MDA5 is particularly important for picornaviruses such as CVB3^163,178,181^ (Fig. 4).

#### Signaling architecture

RIG-I and MDA5 contain tandem N-terminal CARD domains, a helicase domain and a C-terminal RNA-binding domain (Fig. 4). Viral RNA binding promotes conformational rearrangements that expose CARD domains and enable CARD–CARD interactions with MAVS, triggering downstream activation of IRF3/IRF7 and NF-κB, thereby inducing type I IFN and proinflammatory cytokines^182–185^. MAVS knockout studies confirm the central role of the RLRs–MAVS axis in antiviral immunity^186,187^. LGP2, a related helicase lacking CARD domains, can modulate the signaling of RLRs, although its precise mechanisms remain incompletely defined^188^.

#### Evidence in VM models

In EMCV infection, MDA5 or MAVS deficiency results in markedly increased viral titers in the heart early after infection^178,187^. For CVB3, two studies implicate the MDA5–MAVS axis in antiviral defense^189,190^. Both reported reduced IFN-α; however, divergent effects on IFN-β and downstream ISGs have been reported across tissues, underscoring that RLRs outputs can be cell and organ specific^190^. Importantly, direct studies assessing post-CVB3 myocarditis outcomes in MDA5- or MAVS-deficient mice remain limited, representing a key gap for future mechanistic work.

In addition to canonical RLRs/TLRs pathways, emerging evidence supports MAVS-independent RNA-sensing circuits in innate immune cells that can shape VM susceptibility.

### Noncanonical RNA sensor PARP9

Our group identified PARP9 as a noncanonical RNA sensor in DCs that contributes to antiviral defense against cardiotropic RNA viruses such as reovirus^27^. PARP9 binds viral dsRNA (approximately 1,100–1,400 bp in reovirus) and triggers recruitment and activation of PI3K–AKT3, independent of MAVS. PI3K/AKT3 signaling promotes phosphorylation/activation of IRF3 and IRF7, leading to robust type I IFN production (Fig. 4). Functionally, PARP9-deficient mice exhibit increased susceptibility to reovirus-induced VM due to impaired type I IFN responses^27^. This pathway illustrates that RNA sensing in VM is broader than the classical TLR/RLR framework and may reveal therapeutically tractable nodes that preserve antiviral type I IFN without excessive inflammation.

### Cytokine signaling and JAK–STAT feedback control

**Fig. 5:**
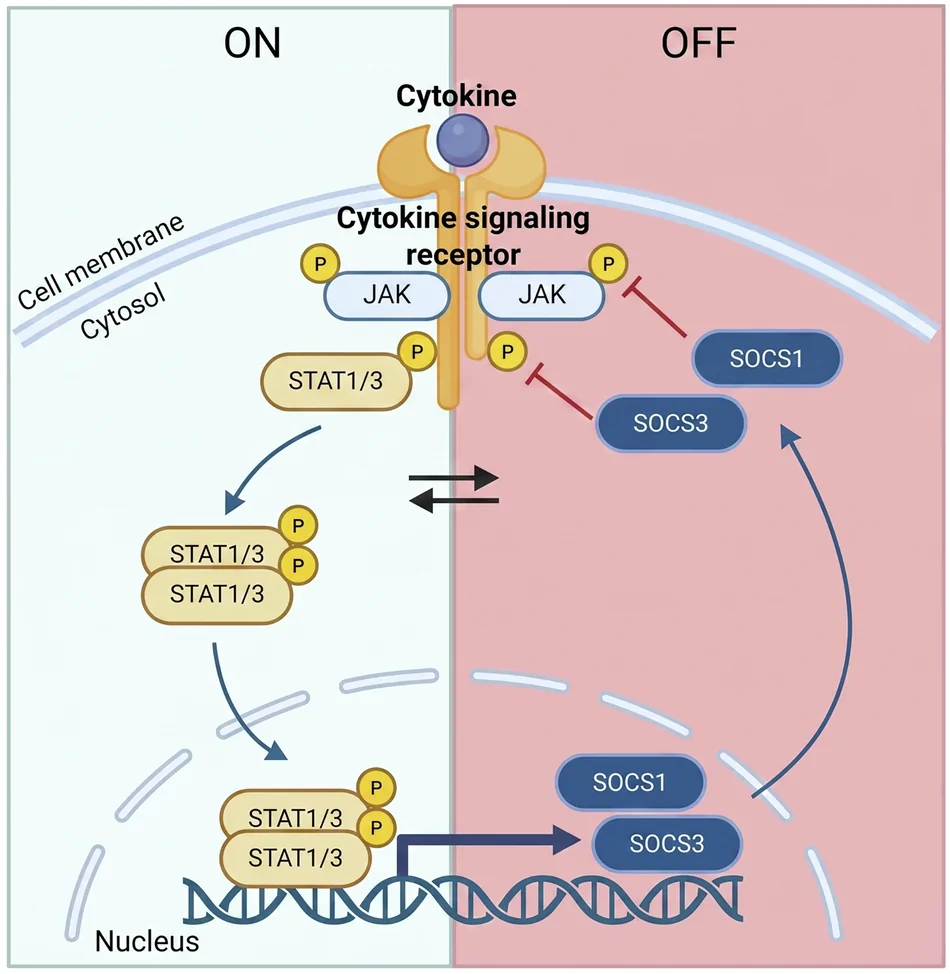
Regulation of the JAK–STAT signaling pathway during VM. Schematic illustration of SOCS-mediated feedback inhibition of the JAK–STAT pathway. Cytokine engagement of cell-surface receptors activates associated Janus kinases (JAKs), leading to phosphorylation and activation of STAT1 and STAT3, which translocate to the nucleus to induce target gene expression, including SOCS1 and SOCS3. SOCS1 directly binds phosphorylated JAK proteins to inhibit kinase activity, whereas SOCS3 associates with phosphorylated tyrosine residues within the cytoplasmic domain of cytokine receptors, thereby blocking further STAT activation. Together, SOCS1 and SOCS3 function as inducible negative regulators that terminate JAK–STAT signaling. Activating signals are shown with blue arrows, whereas negative feedback signals are shown with red arrows.

RNA sensing culminates in cytokine production that coordinates antiviral defense but can also drive immunopathology. In CVB3-induced VM^124,191,192^, multiple cytokines, including IFN-α/β, IFN-γ, IL-6, IL-10 and IL-12, activate JAK–STAT signaling during early disease^193–195^. A critical negative-feedback layer is provided by suppressors of cytokine signaling (SOCS) proteins. Cardiac-specific SOCS1 and SOCS3 transgenic models have been used to interrogate how cytokine feedback affects viral replication and inflammation^196,197^. SOCS1/3 inhibit JAK–STAT signaling by limiting receptor-proximal and STAT phosphorylation events, thereby attenuating cytokine-driven antiviral gene programs^198^. CVB3 infection activates JAK–STAT pathways and induces SOCS1 and SOCS3 expression^197^. Experimental evidence suggests that SOCS1 upregulation can dampen IFN-γ signaling and worsen early viral control in the heart^197,199^ (Fig. 5).

SOCS3 exhibits distinct behavior, in part because it interfaces strongly with gp130-associated cytokine receptors, particularly IL-6 signaling^199,200^. SOCS3 overexpression does not uniformly suppress IFN-γ receptor signaling in cardiomyocytes^196^, and gp130 signaling itself can influence susceptibility to CVB3, as cardiac-specific gp130 deficiency increases vulnerability to infection^196^. Collectively, these data support a model in which cytokine feedback regulators tune the balance between protective antiviral signaling and maladaptive inflammation.

RNA-sensing pathways in VM serve as a double-edged sword: TLR3 and RLR–MAVS signaling are generally protective by limiting viral replication, whereas excessive activation of endosomal sensing and downstream cytokine cascades can amplify inflammatory injury. Noncanonical sensors such as PARP9 reveal additional antiviral circuits that may be leveraged therapeutically. Importantly, because type I IFN and proinflammatory cytokines programs can either protect or injure depending on timing and magnitude, pathway-targeted interventions must consider disease phase and cell-type specificity. In the next section, we extend this framework to DNA-sensing pathways, which are central in DNA virus-associated myocarditis and may also be engaged by self-DNA released during tissue damage, linking nucleic acid sensing to chronic inflammation and remodeling.

## DNA-sensing pathways in antiviral innate immunity during VM

Cardiotropic DNA viruses (for example, adenoviruses, cytomegalovirus, parvovirus B19 and herpesviruses) can trigger VM, yet—compared with RNA virus models (especially CVB3)—the mechanistic evidence linking specific DNA sensors to VM outcomes remains relatively limited. This gap reflects, in part, the historical scarcity of robust, well-standardized DNA virus-induced VM mouse models, which have constrained in vivo dissection of DNA-sensing pathways in the heart. Nonetheless, the conceptual framework is clear: DNA sensors detect viral (and sometimes host-derived) DNA in endosomes, cytosol, or nucleus and activate type I IFN and NF-κB-dependent proinflammatory cytokines^95,201–203^, shaping both antiviral control and immunopathology in the heart. Below, we summarize established and emerging DNA sensors relevant to VM and highlight priorities enabled by new DNA virus-induced VM models.

### Endosomal DNA sensor TLR9

TLR9 is an endosomal DNA sensor that recognizes unmethylated CpG-rich DNA, a molecular pattern enriched in microbial and many viral genomes^204,205^. Upon ligand binding, TLR9 signals primarily through MyD88, activating downstream NF-κB and IRF3/IRF7 pathways that induce proinflammatory cytokines and type I IFN^161,206^ (Fig. 3). TLR9-dependent signaling can influence myocardial function; for example, TLR9 activation has been linked to altered cardiomyocyte contractility^207^. In experimental settings, TLR9 deficiency has been reported to reduce cardiac inflammation, supporting the idea that endosomal DNA sensing can contribute to immunopathology during acute VM and may represent a therapeutic node when inflammation predominates over viral control.

### Cytosolic DNA sensors

#### Cytosolic DNA sensor cGAS

**Fig. 6:**
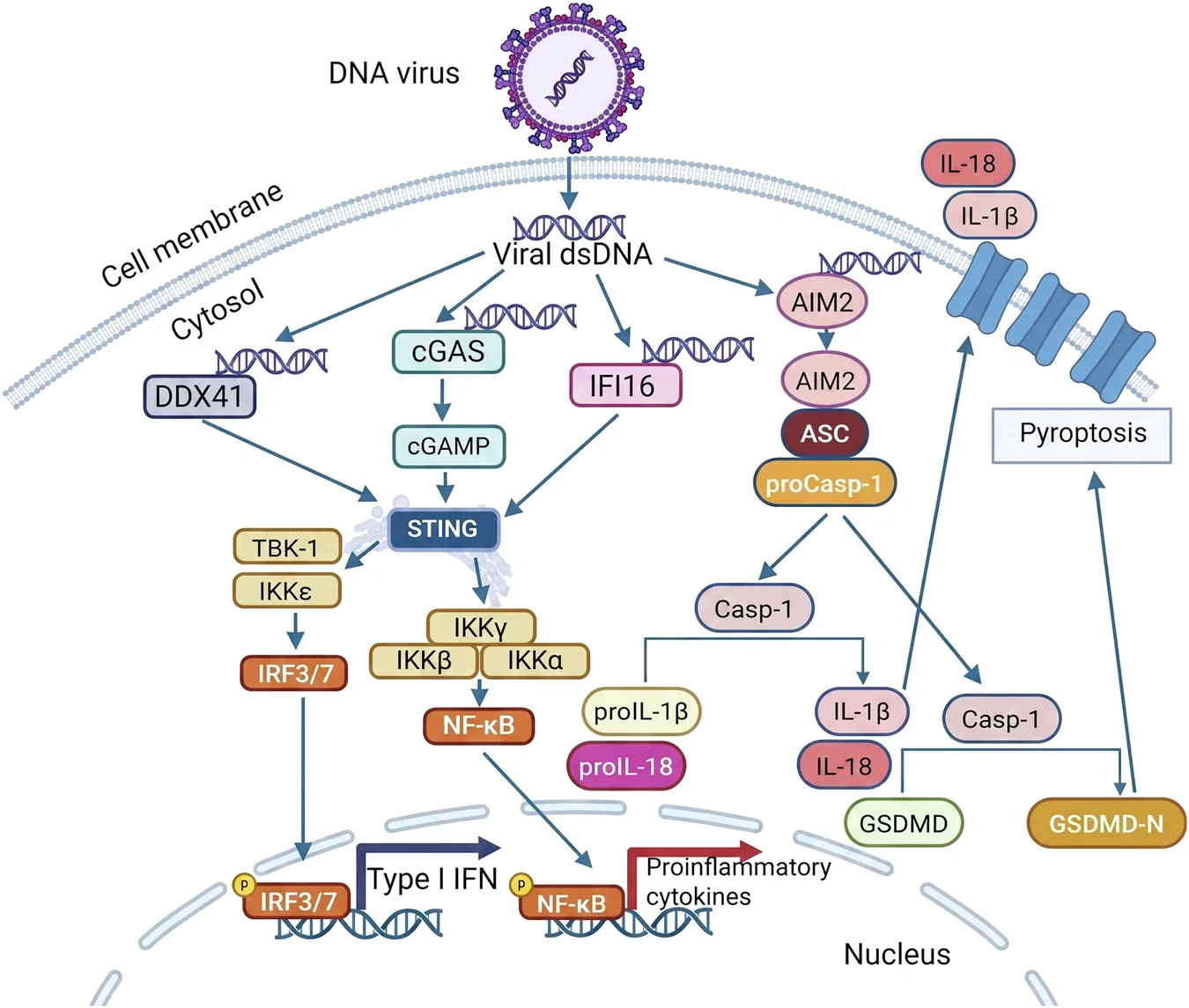
DNA-sensing pathways in antiviral innate immunity during VM. Schematic overview of cytosolic DNA-sensing pathways that drive antiviral innate immune activation in response to cardiotropic DNA viruses. Viral dsDNA is recognized by cyclic GMP–AMP synthase (cGAS), leading to the production of the second messenger cyclic GMP–AMP (cGAMP), which binds and activates the adaptor protein STING. Activated STING recruits and activates TBK1 and the IKK complex, resulting in phosphorylation and activation of IRF3 and IRF7, as well as activation of NF-κB, thereby inducing transcription of type I IFN and proinflammatory cytokines. In parallel, the DNA sensors DDX41 and IFI16 also detect viral dsDNA and signal through STING to engage the same downstream TBK1–IRF and IKK–NF-κB pathways. In addition to STING-dependent signaling, AIM2 functions as an inflammasome-forming DNA sensor: upon binding cytosolic dsDNA, AIM2 recruits the adaptor ASC to assemble the AIM2 inflammasome and activate pro-caspase-1 (proCasp-1). Active caspase-1 (Casp-1) then processes proIL-1β and proIL-18 into mature IL-1β and IL-18 and cleaves gasdermin D (GSDMD) to generate the pore-forming N-terminal fragment (GSDMD-N), promoting cytokine release and inflammasome-associated pyroptotic cell death. Activating signals are shown with blue arrows; type I IFN transcription, which restricts VM, is indicated by a bold blue arrow, whereas transcription of proinflammatory cytokines, which promotes VM, is indicated by a bold red arrow.

Cyclic guanosine monophosphate (GMP)-adenosine monophosphate (AMP) synthase (cGAS) is the best-characterized cytosolic DNA sensor and binds double-stranded DNA (dsDNA) in a sequence-independent manner^208^. DNA engagement activates cGAS enzymatic activity to produce cGAMP, which activates STING on the ER, leading to recruitment of TBK1, phosphorylation of IRF3 and induction of type I IFN, alongside activation of NF-κB-dependent proinflammatory cytokines (Fig. 6). Although most cGAS–STING work has centered on antiviral immunity and sterile inflammation, evidence also implicates STING signaling in inflammatory cardiac phenotypes, and cGAS–STING activity has been discussed in the context of CVB3-associated inflammation^209^. This underscores an important point: even in RNA virus-induced VM, host-derived DNA (for example, from dying cells or damaged mitochondria) can potentially engage DNA-sensing pathways and amplify inflammation. DNA viruses frequently antagonize cGAS–STING. For example, Epstein–Barr virus can induce TRIM29 to target STING for degradation, reducing type I IFN output^94^. Such immune-evasion logic is highly relevant to DNA virus-induced VM, where the balance between viral replication and host inflammation may depend on how efficiently viruses suppress STING signaling in specific cardiac and immune cell compartments.

#### Additional DNA sensors and inflammasome pathways

While cGAS–STING is the dominant cytosolic DNA-sensing axis, several other DNA sensors can shape antiviral and inflammatory outputs^95^. Importantly, for VM, these pathways are plausible and mechanistically compelling, but direct in vivo VM evidence is still comparatively sparse, making them high-priority targets for future DNA virus-induced VM studies.

### DDX41

DDX41 is a DEAD-box helicase that can recognize cytosolic dsDNA (and in some contexts cyclic dinucleotides) and signal through STING, promoting TBK1–IRF3 activation and type I IFN production^210,211^ (Fig. 6). At present, however, evidence linking DDX41 directly to VM is largely inferential and derives mainly from general antiviral innate-immunity models rather than cardiotropic DNA virus-induced VM studies. Our preliminary data show that DCs-specific DDX41-deficient mice develop more severe cardiac inflammation in DNA virus mouse adenovirus type 1 (mAdV)-induced VM mouse model. We will further investigate the underlying mechanisms by which DDX41 regulates DNA virus-induced VM. Testing DDX41 function in DNA virus-induced VM could clarify whether it acts redundantly with cGAS or provides cell-type-specific sensing in cardiac immune niches.

### Interferon gamma inducible protein 16 (IFI16)

IFI16 detects viral dsDNA in the nucleus and can initiate antiviral signaling that converges on STING-dependent type I IFN programs (Fig. 6) and/or inflammasome-related outputs depending on context^212^. Because several cardiotropic DNA viruses replicate in the nucleus, IFI16 is conceptually attractive as an upstream sensor that could link nuclear viral replication to IFN induction and inflammation in infected cardiac cells and resident immune populations.

### Absent in melanoma 2 (AIM2)

AIM2 is a cytosolic dsDNA sensor that triggers inflammation through inflammasome assembly. Upon binding dsDNA, AIM2 recruits the adaptor ASC to form the AIM2 inflammasome, leading to activation of pro-caspase-1. Activated caspase-1 then cleaves proIL-1β and proIL-18 into mature IL-1β and IL-18, and processes gasdermin D (GSDMD) to release its pore-forming N-terminal fragment (GSDMD-N), enabling cytokine secretion and driving inflammasome-associated pyroptotic cell death^213–215^ (Fig. 6). In VM, inflammasome activation promotes maturation and release of IL-1β and IL-18, which can amplify myocardial inflammation by inducing chemokine production, endothelial activation, and recruitment of leukocytes to injured cardiac tissue^216,217^. While AIM2 is classically discussed in DNA-driven inflammation, direct AIM2-specific studies in DNA virus-induced VM remain limited—yet AIM2 is a logical candidate for mediating the ‘inflammation amplification’ component of DNA virus-induced VM, especially when viral control is incomplete or tissue injury is extensive.

### Gap and future directions of the DNA-sensing pathway in VM

Compared with RNA-sensing pathways that have been extensively dissected in CVB3 and other RNA virus-induced VM models, DNA-sensing mechanisms in VM remain underexplored. Progress has been limited largely by the historical lack of robust, reproducible DNA virus-induced VM mouse models, which has constrained in vivo testing of candidate DNA sensors across disease stages and relevant cardiac cell types.

To address this gap, we have recently established mAdV-induced VM and murine cytomegalovirus-induced VM mouse models. These platforms provide a timely opportunity to systematically define how DNA-sensing pathways regulate VM in a phase- and cell type-dependent manner. In particular, DNA sensors—including TLR9, cGAS and emerging axes such as DDX41, IFI16 and AIM2—offer a mechanistic bridge linking cardiotropic DNA viruses (and potentially injury-released host DNA) to downstream type I IFN and proinflammatory cytokines programs. Importantly, the same pathways that support antiviral defense may also fuel immune-mediated myocardial injury and remodeling when excessive or prolonged.

Moving forward, these newly developed DNA virus-induced VM models should enable rigorous dissection of DNA-sensing circuitry and clarify when and where DNA sensing is protective versus pathogenic, thereby guiding pathway-informed therapeutic strategies for DNA virus-induced VM.

## Biomarkers, therapeutic strategies and clinical trials targeting innate immune pathways in VM

Although most patients with VM are treated with supportive heart-failure therapy, growing mechanistic insights into PRRs signaling, type I IFN and proinflammatory cytokines programs, cytokine networks, inflammasome activation and cardiomyocyte stress pathways have enabled more rational biomarker development and pathway-directed interventions in both preclinical models and early clinical studies. A consistent theme across translational efforts is that therapeutic benefit depends on etiology (virus-positive versus virus-negative inflammatory cardiomyopathy) and disease phase (acute viral injury versus subacute immune-mediated damage versus chronic remodeling). Below, we integrate clinically actionable biomarkers with representative therapeutic strategies and ongoing/previous clinical trials (Table 3).

**Table 3:** Selected clinical trials of pathway-targeted therapies and biomarker strategies in myocarditis including VM.

Pathway/mechanism	Intervention	Target population	Clinical trial
Antiviral immunomodulation (type I IFN axis)	Interferon-β-1b	VM/viral cardiomyopathy spectrum (etiology dependent)	NCT00185250
Innate cytokine blockade (IL-1 pathway)	Anakinra (IL-1 receptor antagonist)	Acute myocarditis (hospitalized symptomatic patients; not always viral-stratified)	NCT03018834
Innate cytokine blockade (IL-1 pathway, pediatric)	Anakinra	Pediatric acute myocarditis	NCT07371689
Broad anti-inflammatory therapy (microtubule/inflammasome-linked)	Colchicine	Myopericarditis/myocarditis spectrum (variable definitions)	NCT06158698
Autoimmunity / prognostic biomarkers (anti-heart autoantibodies)	Anti-β1AR and anti-L-CaC antibodies	VM; biomarker-based risk stratification for outcomes	NCT06742008

### Biomarkers for diagnosis, patient stratification and mechanism-guided therapy

#### Core clinical biomarkers (injury and hemodynamic stress)

In clinical practice, myocarditis is suspected in patients presenting with chest pain, arrhythmia, acute heart failure or cardiogenic shock, frequently after a viral prodrome. Cardiac troponins reflect cardiomyocyte injury, while BNP/NT-proBNP indicate myocardial wall stress and correlate with the severity of ventricular dysfunction. Systemic inflammatory markers, such as C-reactive protein, can support an inflammatory phenotype but lack specificity^218^.

#### Imaging biomarkers and tissue characterization

Cardiac magnetic resonance imaging (MRI) provides noninvasive tissue-level biomarkers of inflammation and injury, including edema (T2-based measures) and injury/fibrosis (late gadolinium enhancement; T1 mapping/extracellular volume). These measures aid diagnosis, risk stratification and follow-up of myocardial recovery or remodeling^5^.

#### Etiology stratification biomarkers: viral persistence versus virus-negative inflammation

Because immunosuppression can be detrimental during active viral replication, EMB with histology/immunostaining and viral genome testing (PCR/RT–PCR) remains the most direct approach to classify myocarditis as virus-positive versus virus-negative inflammatory cardiomyopathy. This stratification is clinically consequential: virus-positive disease may benefit from antiviral/immunomodulatory approaches, whereas virus-negative inflammatory cardiomyopathy can respond to immunosuppressive regimens in selected settings.

#### Liquid biopsy for real-time etiologic and injury stratification

A practical barrier to mechanism-guided therapy is the difficulty of rapidly distinguishing persistent viral infection from virus-negative sterile inflammation. EMB remains the reference approach when tissue-level diagnosis is needed, but less invasive liquid biopsy strategies may complement EMB and cardiac MRI. Circulating viral genomes, viral RNA, microRNAs, cell-free DNA (cfDNA) and cell-free RNA can potentially provide longitudinal information about viral persistence, cardiomyocyte injury, immune-cell activation and multiorgan involvement. Recent work using cfDNA methylation signatures showed that plasma cfDNA can reflect organ-specific injury and may help identify early multiorgan damage in fulminant myocarditis, while systematic reviews have highlighted both microRNAs and cfDNA as candidate liquid biopsy biomarkers in myocarditis and inflammatory DCM^219,220^. These approaches are not yet replacements for EMB, but they could support real-time risk stratification, monitoring of treatment response, and enrichment of clinical trials by identifying patients whose disease is dominated by viral persistence versus immune-mediated injury.

#### Mechanistic immune signatures and molecular markers

Mechanistic biomarker development is accelerating. In COVID-19–associated VM, transcriptomic analysis demonstrated enrichment of JAK–STAT3, inflammatory and IFN-response gene sets compared with controls. In that work, B2M and C3 were proposed as diagnostic markers and linked to immune programs consistent with activation of NK cells and DCs, illustrating how innate immune pathway activity can yield clinically informative biomarker candidates^221^.

#### Host genomics and multi-omics for precision risk stratification

Host genetic susceptibility is an important but under integrated component of precision medicine in VM^222^. Variants in inherited cardiomyopathy genes can modify the clinical course of myocarditis and influence risk for arrhythmias, ventricular dysfunction and progression to DCM. A population-based study of acute myocarditis found enrichment of pathogenic or likely pathogenic variants in cardiomyopathy-associated genes compared with controls, supporting overlap between myocarditis and inherited cardiomyopathy biology^223^. Pediatric myocarditis with a DCM phenotype can also harbor pathogenic variants in cardiomyopathy disorder genes, with possible additional contribution from immune-related variants^224^. These observations suggest that whole-exome or whole-genome sequencing, interpreted together with family history, cardiac MRI, viral testing and immune signatures, could help to identify patients at high risk for persistent dysfunction or DCM. In parallel, polygenic and genome-wide association approaches in DCM are beginning to reveal broader genetic architecture that may inform risk prediction and pathway discovery^225^. For VM, the most informative precision strategy will probably combine host genomics with transcriptomics, proteomics, metabolomics, serology and spatial immune profiling to define patient-specific inflammatory set points and recovery trajectories.

#### Artificial intelligence-driven predictive models and foundation models

The increasing use of single-cell transcriptomics, spatial transcriptomics, proteomics, viromics and longitudinal clinical imaging creates datasets that are too complex for traditional biomarker analysis alone. Artificial intelligence-driven models can integrate multimodal data to identify disease states, infer cell–cell communication and predict trajectories from acute VM to chronic DCM or heart failure. Foundation models trained on large single-cell atlases, such as scGPT, illustrate how generative pretrained models can support cell-type annotation, batch integration, perturbation prediction and gene-network inference^226^. In VM, analogous adaptive biohealth models could integrate cardiac immune-cell states, viral burden, host genetic risk, liquid biopsy markers and clinical variables to forecast who will recover, who will develop chronic inflammation and who may benefit from antiviral immunomodulation versus anti-inflammatory therapy. Such models must be prospectively validated and interpreted cautiously, but they provide a forward-looking framework for transforming mechanistic immune profiling into clinically actionable decision support.

#### Autoantibodies and remodeling risk

Autoantibodies against cardiac targets are currently being evaluated as biomarkers of risk for progression from VM to DCM. In particular, ClinicalTrials.gov NCT06742008 is assessing anti-β1-adrenergic receptor (anti-β1AR) and anti-L-type Ca²^+^ channel (anti-L-CaC) antibodies as prognostic biomarkers after acute VM (Table 3).

Collectively, these biomarkers define who has VM, how active injury/inflammation is and whether disease biology is dominated by viral persistence, innate inflammatory amplification or immune dysregulation, thereby informing phase-appropriate therapeutic choices.

### Preclinical therapeutic strategies targeting innate immune pathways

#### Antiviral immunomodulation (type I IFN axis)

Type I IFN is central to restricting cardiotropic viruses and shaping downstream immunity. In settings of viral persistence or chronic viral cardiomyopathy, augmenting antiviral type I IFN responses—rather than suppressing inflammation indiscriminately—has a strong mechanistic rationale and has informed clinical translation (see below).

#### Cytokine pathway targeting and JAK–STAT signaling

Cytokines that signal via JAK–STAT contribute to both antiviral defense and inflammatory injury. The COVID-19 myocarditis transcriptomic data highlighting JAK–STAT3 enrichment^221^ emphasize the clinical relevance of this axis and support ongoing exploration of cytokine-network modulation. A key challenge remains in identifying the appropriate phase and patient subset in which cytokine pathway modulation improves outcomes without impairing antiviral control.

#### Inflammasome inhibition (NLRP3 as an innate inflammatory amplifier)

Inflammasomes provide a mechanistic bridge between nucleic acid sensing/cellular stress and inflammatory cytokine maturation. The NLRP3 inflammasome is particularly attractive as a drug target because it amplifies both IL-1-family signaling and downstream inflammatory cascades. The small-molecule inhibitor MCC950 selectively inhibits NLRP3 activation and reduces inflammatory output, providing a proof of concept that inflammasome blockade is therapeutically tractable^227,228^. These insights have motivated interest in NLRP3-centered strategies in VM-relevant contexts, including models related to CVB3 and inflammatory cardiac injury associated with SARS-CoV-2^216,227^. Conceptually, inflammasome inhibition is most likely to be beneficial when immune-mediated injury dominates or when excessive innate amplification drives heart damage.

#### Extracellular vesicle (EV)-based immunomodulation (platelet-derived EV therapeutics)

A distinct emerging strategy is the use of EVs as biologic immunomodulators capable of delivering complex cargos that reshape immune and repair programs without broad immunosuppression. In a CVB3-induced VM mouse model, reconstituted human platelet-derived EVs reduced myocarditis severity, supporting the feasibility of a stable EV formulation with therapeutic activity^229^. More recently, the therapeutic effects of platelet-derived EV preparations were shown to correlate with their biomolecular content, highlighting cargo-informed optimization and the potential for EV product ‘quality control’ metrics linked to efficacy^230^. Together, these studies position platelet-derived EVs as a scalable immunomodulatory platform for VM.

#### Cardiomyocyte-intrinsic stress pathways (TRIM29–PERK axis and PERK inhibition)

In addition to immune-cell-centered interventions, cardiomyocyte stress signaling can serve as a tractable therapeutic target because it integrates viral replication, regulated cell death and inflammatory amplification. In our recent work, cardiotropic viruses induced TRIM29, which promoted PERK-driven ER stress, apoptosis and reactive oxygen species, thereby facilitating viral replication in cardiomyocytes and causing the pathogenesis of VM^26^. Importantly, pharmacologic inhibition with the PERK inhibitor GSK2656157 mitigated VM, improving cardiac function, enhancing antiviral responses and curbing inflammatory and immunosuppressive programs by disrupting the TRIM29–PERK connection in CVB3-induced VM mouse model^26^. These findings support ER-stress signaling—specifically PERK—as a pathway-defined target that may be particularly relevant when cardiomyocyte-intrinsic injury and immune suppression coexist.

#### Immune balance and T_reg_-supportive circuits (linking innate inflammation to adaptive control)

Translation in VM increasingly involves interventions that reshape the innate–adaptive interface. T_reg_ cells can restrain damaging inflammation and promote repair in myocarditis models. Papageorgiou et al. identified thrombospondin-2 (TSP-2) as an immunomodulatory factor linked to T_reg_-associated protection in CVB3-induced VM, and subsequent studies further supported the therapeutic concept of leveraging TSP-2/T_reg_ mechanisms to mitigate cardiac inflammation and hyperinflammatory injury^141^. These studies have reinforced a broader principle: therapies that dampen innate amplification while preserving antiviral effector function may succeed when they also restore adaptive immune regulation.

Across preclinical VM studies, therapeutic success most often reflects phase-appropriate dampening of injurious innate amplification (for example, IL-1/NLRP3) or targeted correction of pathogenic host programs (for example, EV immunomodulation and cardiomyocyte ER-stress inhibition), while avoiding suppression of indispensable early antiviral defenses.

### Clinical trial landscape: mechanism-guided strategies and practical principles

Clinical translation should be framed with caution because VM is etiologically heterogeneous and clinical evidence for many pathway-targeted interventions remains limited. The strongest mechanistic rationale does not always equal proven clinical benefit. Therefore, therapeutic discussion should distinguish (1) preclinical proof of concept, (2) small clinical studies or selected biopsy-defined cohorts and (3) ongoing randomized trials. In practice, treatment decisions should consider viral positivity, disease phase, severity, ventricular function, arrhythmic risk and whether inflammation is dominated by ongoing viral replication, innate cytokine amplification or autoimmune-like injury. This staged framework is intended to guide hypothesis generation and trial design rather than to imply that all pathway-directed therapies are ready for routine VM treatment.

Clinical evidence so far supports two practical principles for mechanism-guided therapy^30,231^ (Table 3).

### Principle 1: Virus-positive persistence may benefit from antiviral immunomodulation; indiscriminate immunosuppression may be harmful if viral replication persists

Biopsy-characterized cohorts of chronic viral cardiomyopathy have motivated IFN-based strategies aiming to reduce viral persistence and improve function. In Table 3, interferon-β-1b is listed as an antiviral immunomodulatory approach (NCT00185250). This therapeutic logic is consistent with a broader strategy: treat ongoing viral presence with targeted antiviral immunomodulation rather than broad immunosuppression.

### Principle 2: Virus-negative inflammatory cardiomyopathy may respond to immunosuppression, highlighting the need for etiologic classification

In virus-negative inflammatory cardiomyopathy (inflammation present but viral genome testing negative), immunosuppressive regimens (for example, prednisone plus azathioprine in selected settings) have shown durable benefit in prior studies. This underscores why EMB plus viral testing, when feasible, remains central to patient selection for immunosuppression.

### Emerging trials targeting innate inflammatory amplifiers: IL-1 pathway blockade

Targeting the IL-1 pathway reflects growing interest in phase-appropriate immunomodulation in acute myocarditis. Anakinra (IL-1 receptor antagonist) is being evaluated in acute myocarditis (NCT03018834) and in pediatric acute myocarditis (NCT07371689). These efforts align mechanistically with the concept that IL-1-family signaling represents an actionable ‘innate amplifier’ downstream of PRRs and inflammasome activation (Table 3).

### Broad anti-inflammatory strategies with innate links: colchicine

Colchicine has been tested in myocarditis/myopericarditis spectrum populations (NCT06158698). While mechanisms are pleiotropic, colchicine can attenuate inflammatory signaling and has been linked to microtubule-dependent pathways relevant to inflammasome activity, making it a pragmatic anti-inflammatory candidate in selected inflammatory phenotypes (Table 3).

### Biomarker-focused clinical trials: autoantibody risk stratification

NCT06742008 evaluates whether anti-β1AR and anti-L-CaC antibodies predict progression to DCM after acute VM—an example of biomarker development aimed at improving risk prediction and enabling targeted follow-up or trial enrichment (Table 3).

## Conclusions and future perspectives

VM remains a potentially life-threatening inflammatory heart disease that disproportionately affects children and young adults and can culminate in DCM, progressive heart failure, malignant arrhythmias and sudden death. Among cardiotropic pathogens, CVB3 has been a prototypic trigger and an enduring experimental platform that has clarified how direct viral cytotoxicity intersects with host immunity to drive myocardial injury. Across disease phases, both innate and adaptive immune responses shape outcomes: early antiviral programs can be cardioprotective by limiting viral replication, whereas sustained or dysregulated inflammation can amplify tissue damage and promote adverse remodeling. Emerging data further suggest that VM pathogenesis may not be confined to the heart, but can be influenced by peripheral immunologic ‘set points’, including sex-dependent differences and the intestinal commensal microflora—particularly relevant because the gut is a primary site of replication for enteric viruses such as CVB3. These observations raise the possibility that targeting commensal–host interactions and mucosal immunity could modulate downstream systemic responses and ultimately reshape cardiac immunopathology in VM.

Looking forward, translation will increasingly depend on mechanism-guided precision rather than ‘one-size-fits-all’ immunosuppression. A practical framework for next-generation VM management begins with etiologic classification whenever feasible: distinguishing virus-positive from virus-negative inflammatory cardiomyopathy using EMB with viral genome testing is clinically consequential because it informs whether antiviral immunomodulation or immunosuppression is more appropriate. In parallel, clinicians and trialists must stage the disease phase, recognizing that early PRRs/type I IFN signaling and other antiviral pathways may be protective, whereas later excessive innate amplification—such as sustained cytokine programs and IL-1/inflammasome activation—may drive immunopathology, ventricular remodeling and progression to DCM. This phase-aware approach helps to reconcile why interventions that blunt inflammation may be beneficial in some contexts yet harmful when viral replication persists, and it highlights the need to time immunomodulation to the dominant biology.

Finally, achieving true precision will require integrating mechanistic biomarkers with targeted therapy to enrich clinical trials and personalize care. Multi-omic and immune-profiling efforts are beginning to define actionable signatures that reflect pathway activity—such as type I IFN/JAK–STAT programs, IL-1/inflammasome surrogates and immune-cell activation patterns. For example, transcriptomic profiling in COVID-19-associated VM identified enrichment of JAK–STAT3, inflammatory and IFN-response gene sets and proposed B2M and C3 as candidate diagnostic markers, illustrating how mechanistic profiling can inform stratification and therapeutic targeting. In this biomarker-informed paradigm, therapies can be matched to pathway and timing: IFN-based strategies for virus-positive persistence; IL-1 pathway blockade and potentially NLRP3-targeted approaches (for example, MCC950-class inhibitors) for innate inflammatory amplification; and immune-regulatory strategies that restore balance at the innate–adaptive interface (including T_reg_-supportive mechanisms such as TSP-2-associated cardioprotection) during subacute immune-mediated injury. Together, these directions—gut–heart immune axis biology, etiologic and temporal stratification, and biomarker-guided pathway targeting—should accelerate the development of effective, phase-appropriate interventions that prevent progression from acute VM to DCM and heart failure.
